# Are Mental Health Effects of Internet Use Attributable to the Web-Based Content or Perceived Consequences of Usage? A Longitudinal Study of European Adolescents

**DOI:** 10.2196/mental.5925

**Published:** 2016-07-13

**Authors:** Sebastian Hökby, Gergö Hadlaczky, Joakim Westerlund, Danuta Wasserman, Judit Balazs, Arunas Germanavicius, Núria Machín, Gergely Meszaros, Marco Sarchiapone, Airi Värnik, Peeter Varnik, Michael Westerlund, Vladimir Carli

**Affiliations:** ^1^ National Centre for Suicide Research and Prevention of Mental Ill-Health Department of Learning, Informatics, Management and Ethics Karolinska Institutet Stockholm Sweden; ^2^ Department of Psychology Stockholm University Stockholm Sweden; ^3^ Vadaskert Child Psychiatry Hospital and Outpatient Clinic Budapest Hungary; ^4^ Institute of Psychology Eötövös Loránd University Budapest Hungary; ^5^ Clinic of Psychiatry Faculty of Medicine Vilnius University Vilnius Lithuania; ^6^ Skylark Health Research Ltd London United Kingdom; ^7^ Semmelweis University School of Ph.D. Studies Budapest Hungary; ^8^ Department of Medicine and Health Science University of Molise Campobasso Italy; ^9^ National Institute for Health, Migration and Poverty Rome Italy; ^10^ Estonian-Swedish Mental Health and Suicidology Institute Tallinn Estonia; ^11^ Tallinn University Tallinn Estonia; ^12^ Department of Media Studies Stockholm University Stockholm Sweden

**Keywords:** problematic Internet use, addictive behavior, Internet, mental health, adolescent health, longitudinal study

## Abstract

**Background:**

Adolescents and young adults are among the most frequent Internet users, and accumulating evidence suggests that their Internet behaviors might affect their mental health. Internet use may impact mental health because certain Web-based content could be distressing. It is also possible that excessive use, regardless of content, produces negative consequences, such as neglect of protective offline activities.

**Objective:**

The objective of this study was to assess how mental health is associated with (1) the time spent on the Internet, (2) the time spent on different Web-based activities (social media use, gaming, gambling, pornography use, school work, newsreading, and targeted information searches), and (3) the perceived consequences of engaging in those activities.

**Methods:**

A random sample of 2286 adolescents was recruited from state schools in Estonia, Hungary, Italy, Lithuania, Spain, Sweden, and the United Kingdom. Questionnaire data comprising Internet behaviors and mental health variables were collected and analyzed cross-sectionally and were followed up after 4 months.

**Results:**

Cross-sectionally, both the time spent on the Internet and the relative time spent on various activities predicted mental health (*P*<.001), explaining 1.4% and 2.8% variance, respectively. However, the consequences of engaging in those activities were more important predictors, explaining 11.1% variance. Only Web-based gaming, gambling, and targeted searches had mental health effects that were not fully accounted for by perceived consequences. The longitudinal analyses showed that sleep loss due to Internet use (ß=.12, 95% CI=0.05-0.19, *P*=.001) and withdrawal (negative mood) when Internet could not be accessed (ß=.09, 95% CI=0.03-0.16, *P*<.01) were the only consequences that had a direct effect on mental health in the long term. Perceived positive consequences of Internet use did not seem to be associated with mental health at all.

**Conclusions:**

The magnitude of Internet use is negatively associated with mental health in general, but specific Web-based activities differ in how consistently, how much, and in what direction they affect mental health. Consequences of Internet use (especially sleep loss and withdrawal when Internet cannot be accessed) seem to predict mental health outcomes to a greater extent than the specific activities themselves. Interventions aimed at reducing the negative mental health effects of Internet use could target its negative consequences instead of the Internet use itself.

**Trial Registration:**

International Standard Randomized Controlled Trial Number (ISRCTN): 65120704; http://www.isrctn.com/ISRCTN65120704?q=&filters=recruitmentCountry:Lithuania&sort=&offset= 5&totalResults=32&page=1&pageSize=10&searchType=basic-search (Archived by WebCite at http://www.webcitation/abcdefg)

## Introduction

Depression and anxiety are two of the most prevalent psychiatric disorders among adolescents [1-3], and suicide, which is often closely related to these disorders, is the second leading cause of death in the world for 15- to 29-year olds (after traffic accidents) [4]. Over the past decade, there has been a growing interest and concern about how adolescents’ mental health and emotional development are affected by their Internet use. Almost 80% of the European population are Internet users, with percentages above 90% in some countries [5], and with the increasing use of smartphones, more and more individuals have instant and continuous access to the Internet. Over 90% of 16- to 24-year olds in Europe regularly use the Internet at least weekly, a percentage that is higher than for any other age group [6]. Although it is difficult to measure exactly how much time is spent on the Internet, most young people access the Internet on a daily basis, and the Internet has become a well-integrated part of their lives. This has led to changes in how people live their lives and how they construct and maintain social relations and self-identities, seek information, and enjoy entertainment.

A major line of research has linked mental health problems to what has been termed problematic Internet use (or pathological or compulsive Internet use), which is often conceptualized as an impulse control disorder similar to gambling addiction and other behavioral addictions. The most used and validated measure of problematic Internet use, the Internet Addiction Test (IAT) [7], was constructed through an Internet use-specific reformulation of the Diagnostic and Statistical Manual of Mental Disorders Fourth Edition (DSM-4) diagnostic criteria for Pathological Gambling Disorder (for a review of problematic Internet use measurements, see [8]). As such, this screening instrument measures compulsive aspects of Internet use resulting in clinical impairment or distress (eg, feeling preoccupied with the Internet; inability to control or reduce Internet use; feeling moody or depressed when attempting to stop or reduce Internet use; staying online longer than intended; lying about excessive Internet use, and so forth). However, there is no standardized way of classifying problematic Internet use because measurements, cutoffs, and classification procedures vary between studies [8-9]. These differences in diagnostic procedures aside, numerous studies have found problematic Internet use to correlate with DSM Axis I disorders, mainly depression but also social phobia and anxiety, substance use, attention-deficit hyperactivity disorder, and certain personality variables such as hostility [10-13]. The putative mechanism by which problematic Internet use affects mental health is partly related to the excessive time spent on Web-based activities, which results in a neglect of protective offline activities such as sleep, physical exercise, school attendance, and offline social activities, and partly related to symptoms of withdrawal when those activities cannot be accessed [9,14].

Studies show that the problematic aspects of certain individuals’ Internet use are restricted to one or a few specific Web-based activities (eg, gaming or social media use), whereas other activities are nonproblematic [15-17]. Although there is some recent evidence that the factor structure of the IAT [7] is consistent across measuring problematic engagement in specific activities such as gambling and gaming [18], this has led to a differentiation between generalized problematic Internet use and specific forms of problematic Internet use. For example, as most Internet-use research has focused on problematic Web-based gaming, and as many studies have found an association between gaming and severe mental health symptomology, this is the only specific form of problematic Internet use that has been considered for inclusion in DSM-5, whereas generalized problematic Internet use and other specific forms have not [9,19].

It is thus important to differentiate between activities when investigating the mental health effects of Internet use. In some cases, it could be important because the activity in question is prone to becoming addictive, such as Web-based gambling (eg, Web-based poker, sports betting, casino spins) [20-23]. In other cases, it might be important because the content itself may impact mental health by producing specific emotional, cognitive, or behavioral reactions. For example, 1 study on social media use suggests that passive consumption of social content increases feelings of loneliness, whereas direct communication with friends does not [24]. Another example is performing information searches. Studies show that young people, including those with mental health problems, often perform targeted searches related to their physical and mental health [25-27]. Depending on what information they find, this type of behavior could probably have both negative and positive outcomes. Website content that promotes self-destructive behaviors or self-harm may be of particular concern. Furthermore, adolescents perform increasing amounts of school work using the Internet, and as academic performance is usually associated with better mental health [28], using the Internet for such purposes might be predictive of positive mental health rather than what would be expected from a problematic Internet use perspective [29,30]. Other research has shown that certain types of games (eg, massively multiplayer online role-playing games) and certain motives for playing those games (in-game achievement, socializing, immersion, relaxation, and escapism) are predictive of mental health problems and problematic gaming [31-33]. Although the majority of previous research is correlational, it suggests that Internet use can impact mental health either through the activity or content that is used or through delayed consequences that follow the use of the Internet.

This study aimed to investigate how adolescents’ mental health is predicted by time spent on the Internet and their level of engagement in 7 types of Internet activities: social media use, gaming, gambling, pornography viewing, newsreading or watching, activities related to school or work, and targeted information searches that are not related to school or work. Second, the study also tested whether these effects would be sustained or accounted for by perceived consequences of using those Web-based activities. We investigated the impact of both negative consequences (eg, withdrawal, sleep loss) and positive consequences (eg, enjoyment, finding new friends). In addition to performing these analyses on cross-sectional data, we also tested whether these effects would predict changes in mental health over a period of 4 months.

## Methods

### Study Design

Data were collected as a part of the Suicide Prevention through Internet and Media Based Mental Health Promotion (SUPREME) trial (Current Controlled Trials ISRCTN65120704). The study was carried out by collaborating mental health research centers in Estonia, Hungary, Italy, Lithuania, Spain, Sweden, and the United Kingdom. As part of this project, a randomized controlled longitudinal study was carried out in 2012-2013 to evaluate a Web-based mental health intervention website, which was tested in a randomly selected sample of adolescents in a selected area of these countries. Inclusion criteria of the schools were: (1) the school authority agrees to participate; (2) the school is a state school (ie, not private); (3) the school contains at least 100 pupils within the age range of 14-16; (4) the school has more than 2 teachers for pupils aged 15 years; (5) no more than 60% of pupils are of either gender. Participants were cluster randomized, based on school affiliation, into either a full-intervention condition (with access to the intervention website) or a minimal-intervention control group (without access to the intervention website), and were administered an evaluation questionnaire at baseline and at 2 and 4 months of follow-up. The questionnaire included questions about their Internet habits, mental health and suicidal behaviors, and other variables relevant to the evaluation. This study did *not* aim to evaluate any effects of the Web-based intervention but instead explored Internet-related risk factors for mental health problems.

### Participants

Subjects were registered pupils of state schools randomly selected from a predefined area in each country: West Viru County (Estonia), Budapest (Hungary), Molise (Italy), Vilnius city (Lithuania), Barcelona city (Spain), Stockholm County (Sweden), and eastern England (the United Kingdom). Eligible state schools in these areas were randomly arranged into a contact order, the order in which schools were contacted and asked to participate. If a school declined, the next school on the list was contacted. If a school accepted participation, a team of researchers went to the school and presented the background, aims, goals, and procedures of the study to the pupils verbally and through consent forms. As the study procedure included screening for suicidal adolescents, participation was not completely anonymous, but participants’ identities were encrypted in the questionnaire. Written consent was obtained from all pupils who agreed to participate (as well as from one or both parents according to ethical regulations in the region). The study was approved by ethics committees in all participating countries.

The sampling procedure resulted in a total number of 2286 adolescents participating at baseline (Estonia=3 schools, 416 participants; Hungary=6 schools, 413 participants; Italy=3 schools, 311 participants; Lithuania=3 schools, 240 participants; Spain=3 schools, 182 participants; Sweden=9 schools, 337 participants; the United Kingdom=3 schools, 387 participants). Of the participants, 1571 (68.72%) were randomized to the full-intervention group and 715 (31.27%) to the minimal-intervention group. There was a notable dropout rate in the study. In the total sample, the number of subjects that discontinued participation comprised 467 pupils (20.42%) between T1 and T2 and 244 pupils (13.41%) between T2 and T3. Subjects were included in the longitudinal analyses if they had participated at least at T1 and T3, but participation at T2 was not necessary. This resulted in a longitudinal sample of 1544 subjects, with 56% women and a mean age of 15.8 years (standard deviation, SD=0.91 years).

### Internet Use Measures

Measures of Internet behaviors and uses were constructed specifically for this study. This included items that measured the regularity of Internet use (eg, using the Internet once a month vs using it once a week) and the number of hours spent on the Internet on a typical week. Participants were also asked to rate how much time they spend on 7 different activities when using the Internet (socializing, gaming, school- or work-related activities, gambling, newsreading or watching, pornography, and targeted searches that are not related to school or work). Participants rated these activities on a 7-point scale (1=I spend very little or no time doing this; 7=I spend very much time doing this). The last set of items asked participants to rate the self-perceived consequences of engaging in said activities. Participants were asked to rate the extent to which various consequences apply to them, but *only* in relation to those activities that he or she engaged in to a considerable degree (had previously rated as ≥4). The participants rated, on a 7-point scale (1=very seldom or never; 7=very often), the occurrence of the following consequences: “I find new friends”; “I have fun”; “I learn interesting things”; “I stay online longer than intended”; “I chose these activities instead of hanging out with friends (In real life)”; “I stay up late and lose sleep”; “I feel depressed or moody when I have no access to the above mentioned activities”. Participants also rated how their Internet use affected their work performance or school grades (1=my work or grades suffer; 4=not affected at all; 7=my work or grades improve) and whether it was thought to contribute to their life meaning (1=less meaningful; 4=equally meaningful as without them; 7=more meaningful).

For the sake of clarity, we refer to some of these consequences as “positive” (finding new friends; having fun; learning interesting things) because they are outcomes of Internet use that do not necessarily imply addictive behavior and can be expected to lead to better mental health (if at all). We refer to other consequences as “negative” (staying on the Internet longer than intended; choosing Web-based activities instead of offline social activities; staying up and losing sleep; feeling moody when Web-based activities cannot be accessed) because they suggest symptoms of problematic Internet use and can therefore be expected to lead to poor mental health. For example, these negative consequences resemble those included in the IAT [7] and the Internet Gaming Disorder measurement recommendations by Petry et al [9]. Finally, some consequences are considered “bidirectional” (My work or grades improve/suffer; My life becomes less or more meaningful) because subjects could rate them either negatively or positively or indicate no change at all.

### Mental Health Measures

Participants’ levels of depression, anxiety, and stress were assessed by means of the 3 subscales constituting the 42-item version of the *Depression Anxiety Stress Scale* (*DASS-42*) [34]. Each subscale consists of 14 statements that are scored on a 4-point Likert scale according to how much the statement applied to the person over the past week. The scales are designed to measure negative emotional states of depression (dysphoria, hopelessness, devaluation of life, self-deprecation, lack of interest or involvement, anhedonia, and inertia), anxiety (autonomic arousal, skeletal muscle effects, situational anxiety, and subjective experience of anxious affect), and stress or tension (difficulty relaxing, nervous arousal, and being easily upset or agitated, irritable or over-reactive, and impatient). Studies that have investigated the psychometric properties of this scale have reported satisfactory outcomes on reliability and validity measures in healthy and clinical populations [34-37], also when administered over the Internet [38]. However, there have been reports that young adolescents distinguish less between the 3 factors as compared with adults, and correlations among them are typically high [39,40]. The scales demonstrated high internal consistency in the present sample, in terms of Cronbach alpha calculated on the baseline data (depression alpha=.93; anxiety alpha=.89; stress alpha=.91). As some participants did not respond to all scale items, the final score on each scale was calculated by dividing the sum score by the number of items that they had responded. Only participants with 50% missing data or more were excluded. The scales correlated highly with each other (depression × anxiety: *r*=.76; depression × stress: *r*=.79; anxiety × stress: *r*=.78; all *P* values <.001), and the combined 42-item scale demonstrated high internal consistency (alpha=.96). Due to the relatively high intercorrelation between constructs, and to simplify analysis, the 3 scales were combined into a single measure of mental health.

### Procedure

All study procedures took place at the respective schools in classrooms or computer rooms. The questionnaires were administered either in paper and pencil format or using a Web-based survey tool, if the school was able to provide computers for all pupils at time of data collection. The questionnaire contained items used to screen for suicidal adolescents (The Paykel Suicide Scale [41]), and the screening procedure took place within 24 hours after each wave of data collection. Therefore, participation was not completely anonymous; however, subjects’ identities were encrypted using individual “participation codes,” which were written on the questionnaire instead of the participants’ name. The codes were linked to pupil’s identities only to connect data longitudinally and to contact high-risk suicidal adolescents (emergency cases) to offer help. Subjects were defined as emergency cases if they responded that they had seriously contemplated, planned, or attempted suicide within the past 2 weeks. The exact procedure for dealing with risk cases varied between countries and was contingent on the regional ethical guidelines and available help resources. Emergency cases were excluded from the data analysis (n=23). The intervention tested in the SUPREME project was administered after baseline data collection and is described further in Multimedia Appendix 1.

### Data Analysis

Two main analyses were performed in this study: 1 cross-sectional hierarchical multiple regression analysis and 1 longitudinal analysis. The measure of frequency of Internet use was omitted from analysis owing to a ceiling effect (90% of participants reported using the Internet at least once per day). The remaining predictor variables were thus the self-reported number of weekly hours online, the ratings of the 7 activities, and the ratings of the 9 consequences of Internet use. The composite DASS score was the dependent variable in these analyses (tests of statistical assumptions are described in Multimedia Appendix 1). In the cross-sectional regression, Internet behaviors at T1 were used to predict mental health at T1. The longitudinal regression analysis predicted change in overall DASS (the score difference between T1 and T3) by means of change in Internet behaviors. Only the longest follow-up was of interest in this study. Gender, age, and experimental condition were included as control variables in the first model. Time spent on the Internet was added in the second model, activity ratings were added in a third model, and the consequence ratings were added in a fourth model. Further, because participants were instructed to rate perceived consequences only if they performed at least one online activity above the >3 threshold, a minority (n=82; 5%) of subjects whose scores had transcended above or below the threshold between T1 and T3, had incomplete data for the calculation of difference scores. However, sensitivity analyses indicated no statistically significant difference between these subjects and other cases, regarding the average amount of longitudinal change in DASS scores or mean online activity scores.

## Results

### Descriptive Results

DASS-42 scores could be calculated for 2220 participants. Total DASS scores ranged between 0-3 points, where higher scores indicate more mental health problems. The mean baseline scores for males, females and the total sample are presented in Table 1. Females scored significantly higher than males on all mental health measures (Table 1). In the total sample, 1848 participants (83.24%) had a mean DASS score below 1, and 314 (14.1%) had a score between 1 and 1.99, and 58 (2.6%) had a score of 2 or above. There were small but significant differences between the countries in DASS scores (*F*_(6, 2213)_=9.28, η^2^_partial_=.02, *P*<.001). The average change in DASS scores over the 4-month study period was −0.15 (SD=0.42), which indicates a decrease over time. Participants who dropped out of the study between T1 and T3 had somewhat higher baseline DASS scores than adhering participants (mean difference=0.10; *t*_(2218)_=4.068; *P*<.001).

Table 1 also summarizes the average reported time spent on the Internet, activity ratings, and consequence ratings at baseline. The table summarizes that average number of hours spent on the Internet per week was 17.23, with large variation in the sample, and that men had spent slightly more hours on the Internet than women. It was most common for the adolescents to use the Internet for social purposes, followed by school or work, targeted searches, gaming, newsreading or watching, pornography viewing, and gambling, although there were notable gender differences regarding these activities.

**Table 1:** Descriptive results (means and standard deviations) for mental health and Internet use measures at baseline.

Variable^a^	Total	Women	Men	Gender difference^b^		
	M (SD)	M (SD)	M (SD)	*t*	*P*	*d*
Depression	0.52 (0.59)	0.62 (0.64)	0.40 (0.49)	9.15	<.001	0.40
Anxiety	0.48 (0.49)	0.54 (0.51)	0.40 (0.45)	7.02	<.001	0.30
Stress	0.72 (0.60)	0.83 (0.63)	0.57 (0.53)	10.39	<.001	0.37
DASS (total)	0.57 (0.52)	0.67 (0.54)	0.46 (0.45)	9.71	<.001	0.42
Time spent on the Internet	17.12 (17.72)	16.43 (17.04)	17.96 (18.50)	−1.99	.046	−0.09
Socializing	4.94 (1.73)	5.29 (1.62)	4.51 (1.77)	10.80	<.001	0.46
Gaming	3.05 (2.04)	2.03 (1.42)	4.33 (1.98)	−31.95	<.001	−1.33
School or work	3.71 (1.54)	4.01 (1.49)	3.34 (1.52)	10.44	<.001	0.45
Gambling	1.31 (0.98)	1.09 (0.51)	1.58 (1.30)	−12.06	<.001	−0.50
News	2.96 (1.66)	2.93 (1.63)	2.99 (1.69)	−0.83	.41	NS
Pornography	1.73 (1.48)	1.10 (0.55)	2.53 (1.86)	−25.42	<.001	−1.04
Targeted searches	3.28 (1.68)	3.28 (1.68)	3.29 (1.68)	−0.19	.85	NS
Finding friends	3.42 (1.79)	3.40 (1.81)	3.44 (1.76)	−0.45	.65	NS
Learning	4.07 (1.64)	4.02 (1.60)	4.12 (1.68)	−1.35	.18	NS
Having fun	4.66 (1.77)	4.49 (1.73)	4.88 (1.80)	−5.08	<.001	−0.22
Meaningfulness	4.12 (1.22)	4.10 (1.15)	4.14 (1.30)	−0.69	.49	NS
Impact on grades	3.95 (1.24)	3.95 (1.24)	3.93 (1.24)	0.37	.71	NS
Staying on the Internet longer	4.03 (1.86)	4.22 (1.84)	3.79 (1.86)	5.34	<.001	0.23
Prefers Web-based relations	2.14 (1.44)	1.99 (1.38)	2.31 (1.49)	−5.16	<.001	−0.22
Sleep loss	3.07 (1.97)	3.05 (1.98)	3.09 (1.95)	−0.51	.61	NS
Withdrawal (negative mood when inaccessible)	2.25 (1.52)	2.24 (1.54)	2.26 (1.49)	−0.22	.83	NS

^a^Mental health scores (depression, anxiety, stress, DASS total) range between 0 and 3. Time spent on the Internet is measured in hours. All other Internet-related measures range between 1 and 7.

^b^Gender differences were determined through independent samples t-tests; *t*-values, *P* values, and Cohen’s *d* are presented.

### Cross-Sectional Regression Analysis

The cross-sectional hierarchical multiple regression analysis was used to predict DASS scores at T1 by means of Internet use at T1. The first model comprising the control variables (gender, age, experimental condition) was highly significant (*F*_(3, 1683)_=26.40, *P*<.001) and explained *R*^2^_adj_=4.3% of the variance in psychopathology. The second model (time spent on the Internet) contributed significantly to the prediction (*F* change_(1, 1682)_=26.05, *P*<.001) by 1.4%, resulting in a total of *R*^2^_adj_=5.7% explained variance. The third model (relative time spent on activities) contributed significantly to the prediction (*F* change_(7, 1675)_=8.29, *P*<.001) by 2.8%, resulting in a total of *R*^2^_adj_=8.5% explained variance. The fourth model (consequences of Internet use) contributed significantly to the prediction (*F* change_(9, 1666)_=26.80, *P*<.001) by 11.1%. This resulted in a final total of *R*^2^_adj_=19.6% explained variance, 15.3% of which was accounted for by Internet-related factors. The adjusted *R*^2^ continued to increase at each step in the analysis, indicating that the model was not overfitted. There was no indication of problematic collinearity as all variables had a tolerance above 0.5. The results of the regression analysis, including the standardized beta coefficients (ß) for each predictor in each model, are summarized in Table 2.

Table 2 summarizes that gender was the only significant control variable, whereas age and experimental condition were not. The self-reported average number of hours spent on the Internet was a significant predictor of higher DASS scores in models 2 and 3 but not when accounting for consequences of Internet use in the fourth model. The effect size (ß) of individual Web-based activities varied between .05 and .13. Using the Internet for social purposes was a significant predictor of DASS scores in model 3, but not in model 4, suggesting that the risk associated with socializing on the Internet was accounted for by the consequences measured in the study. Web-based gaming followed the opposite pattern, as this activity was not a significant predictor of DASS in model 3 but turned significant in the fourth model. The negative beta value indicates that Web-based gaming was a protective factor associated with mental health. Performing school or work activities on the Internet was also a significant protective factor for psychopathology in the third model but not when accounting for consequences of Internet use. Web-based gambling was a significant risk factor for higher DASS scores in both models 3 and 4. Consuming news content was not significantly associated with DASS in either model. Viewing pornographic content on the Internet was a significant risk factor only in model 3 but not model 4, thus accounted for by consequences of Internet use. Performing targeted searches on the Internet was significantly and strongly positively associated with DASS scores in both models 3 and 4, having the largest effect size of the activities. Regarding consequences of Internet use, finding new friends, learning interesting things, and having fun did not predict DASS scores in model 4. Thus, these “positive” consequences did not seem to act as protective factors. However, Internet use that was perceived to increase life meaning or improve school or work performance was a significant protective factor. The “negative” consequences were more powerful predictors of DASS scores. Although staying on the Internet longer than originally intended was not a significant predictor, the statements “I choose these activities instead of hanging out with friends,” “I stay up late and lose sleep,” and “I feel depressed or moody when I have no access to the above-mentioned activities” were highly significant risk factors, with effect sizes (ß) ranging between .12 and .22.

**Table 2:** Results from the cross-sectional hierarchical multiple regression analysis. Statistics are presented for each predictor variable in each model.

Entered in model no	Predictor variable	Model no^a^	Standardized Beta	95% CI	*t*	*P*	Tolerance
1	(Constant)	1			1.07	.29	
		2			0.41	.68	
		3			0.03	.97	
		4			−0.30	.77	
1	Exp. Condition^b^	1	.00	−0.05 to 0.04	−0.12	.90	1.00
		2	−.01	−0.05 to 0.04	−0.28	.78	1.00
		3	.00	−0.05 to 0.05	0.02	.98	0.99
		4	.02	−.03 to 0.06	0.80	.42	0.98
1	Gender^c^	1	−.21	−0.26 to −0.16	−8.80	<.001	1.00
		2	−.22	-0.26 to −0.17	−9.10	<.001	1.00
		3	−.26	−0.32 to −0.20	−8.03	<.001	0.52
		4	−.22	−0.28 to −0.16	−7.10	<.001	0.51
1	Age	1	−.03	−0.07 to 0.02	−1.04	.30	1.00
		2	−.01	−0.06 to 0.04	−0.39	.69	0.98
		3	.00	−0.05 to 0.05	−0.02	.99	0.97
		4	.01	−0.04 to 0.05	0.31	.76	0.92
2	Time spent on the Internet	2	.12	0.08-0.17	5.10	<.001	0.98
		3	.10	0.05-0.14	3.88	<.001	0.91
		4	.02	−0.03 to 0.07	0.93	.35	0.84
3	Socializing	3	.05	0.00-0.10	2.06	.04	0.90
		4	−.01	−0.06 to 0.03	−0.57	.57	0.78
3	Gaming	3	−.02	−0.07 to 0.04	−0.54	.59	0.64
		4	−.06	−0.12 to −0.01	−2.17	.03	0.57
3	School or work	3	−.05	−0.10 to 0.00	−2.02	.04	0.83
		4	−.03	−0.08 to 0.02	−1.22	.22	0.78
3	Gambling	3	.08	0.03-0.13	3.11	.002	0.89
		4	.05	0.01-0.10	2.30	.02	0.87
3	News	3	.01	−.04 to 0.06	0.50	.62	0.85
		4	.03	−0.02 to 0.07	1.08	.28	0.82
3	Pornography	3	.07	0.01-0.12	2.46	.01	0.72
		4	.02	−0.03 to 0.07	0.78	.44	0.71
3	Targeted searches	3	.13	0.08-0.18	4.94	<.001	0.84
		4	.09	0.04-0.14	3.56	<.001	0.75
4	Finding friends	4	.03	−0.01 to 0.08	1.38	.17	0.79
4	Learning	4	.01	−0.04 to 0.06	0.34	.73	0.67
4	Having fun	4	−.05	−0.10 to 0.00	−1.80	.07	0.71
4	Meaningfulness	4	−.05	−0.10 to −0.01	−2.22	.03	0.90
4	Impact on grades	4	−.07	−0.11 to −0.02	−2.78	.005	0.88
4	Staying on the Internet longer	4	.01	−0.04 to 0.07	0.53	.60	0.66
4	Prefers Web-based relations	4	.12	0.07–0.17	4.74	<.001	0.79
4	Sleep loss	4	.13	0.08-0.19	4.95	<.001	0.65
4	Withdrawal (neg. mood when inaccessible)	4	0.22	0.17-0.27	8.80	<.001	0.76

^a^The model numbers designate which values were obtained when (1) only control variables were analyzed, (2) when time spent over the Internet was added to the model, (3) when Web-based activities were added to the model, and (4) when consequences of Internet use were added to the model.

^b^For experimental condition, the minimal-intervention condition constitutes the reference group.

^c^For gender, females constitute the reference group.

### Longitudinal Regression Analysis

The longitudinal hierarchical multiple regression analysis was used to predict change in overall psychopathology (the score difference between T1 and T3) by means of change in Internet use. There was no indication of problematic levels of collinearity in the model, as all variables had a tolerance value above 0.7. The first model comprising the control variables (gender, age, experimental condition) was not significant (*F*_(3, 981)_ < 1, *P*=.59), and neither was the second model (time spent on the Internet; *F* change_(1, 980)_ < 1, *P*=.95). The third model (relative time spent on activities) contributed significantly to the prediction (*F* change_(7, 973)_=2.25, *P*<.03) by *R*^2^_adj_=0.7% explained variance. This contribution was attributable to news viewing, where an increase in news viewing from T1 to T3 was associated with an increase in DASS scores (ß=.07, 95% CI=0.00-0.13, *P*=.049). All other Web-based activities were nonsignificant (*P*≥ .19) in this model. The fourth model (consequences of Internet use) contributed significantly to the prediction (*F* change_(9, 964)_=3.39, *P*<.001) by 2.1%, resulting in a total of *R*^2^_adj_=2.8% explained variance. News consumption was rendered nonsignificant here (*P*=.13). The contribution of the fourth model was attributable to 2 of the negative consequences. The statements “I stay up late and lose sleep” (ß=.12, 95% CI=0.05-0.19, *P*=.001) and “I feel depressed or moody when I have no access to the above mentioned activities” (ß=.09, 95% CI=0.03-0.16, *P*<.01) were significant predictors in this model. All other predictors were nonsignificant (change in life meaning: *P*=.10; other variables had *P* values above that).

Thus, Internet use that was reported to result in staying up late and losing sleep (“sleep loss”) and to produce negative mood when it could not be accessed (“withdrawal”) were the only variables that consistently predicted longitudinal change in mental health. To further investigate these negative consequences, 2 standard multiple regressions were calculated to predict longitudinal changes in each of these variables by means of changes in time spent on the Internet and the different Web-based activities. The regression model that predicted sleep loss was significant (*F*_(8, 1120)_=5.76, *P*<.001, *R*^2^_adj_=3.3% explained variance) and so was the regression that predicted withdrawal (*F*_(8, 1125)_=11.17, *P*<.001, *R*^2^_adj_=6.7% explained variance). The coefficients from these regressions are summarized in Table 3 and Table 4, respectively. Table 3 summarizes that the strongest predictor for increased sleep loss was a decrease in school or work activities, followed by increased gaming, targeted searching, pornography viewing, and online time in general. Social activities, gambling, and news viewing were not significantly related to change in sleep loss. Table 4 summarizes that the strongest predictors of change in withdrawal were gambling activities, followed by overall time spent on the Internet, pornography viewing, and gaming. Changes in social activities, school or work, news viewing, and targeted searches were not significantly associated with change in withdrawal.

**Table 3:** Results from the multiple regression analysis predicting changes in “sleep loss” by means of change in Internet use.

Predictor variable	Standardized beta	95% CI	*t*	*P*
Constant			0.82	.42
Time spent on the Internet	.07	0.01-0.13	2.25	.03
Socializing	.06	0.00-0.11	1.89	.06
Gaming	.08	0.02-0.14	2.59	.01
School or work	−.10	−0.16 to −0.04	−3.16	.002
Gambling	.01	−0.05 to 0.07	0.36	.72
News	.04	−0.02 to 0.10	1.20	.23
Pornography	.06	0.01-0.12	2.14	.03
Targeted search	.08	0.02-0.14	2.56	.01

**Table 4:** Results from the multiple regression analysis predicting changes in “withdrawal” by means of change in Internet use.

Predictor variable	Standardized beta	95% CI	*t*	*P*
Constant			3.47	.001
Time online	.12	0.06-0.17	3.93	<.001
Socializing	.03	−0.03 to 0.09	1.03	.31
Gaming	.08	0.02-0.13	2.56	.01
School or work	.00	−0.06 to 0.06	−0.03	.97
Gambling	.14	0.08-0.20	4.75	<.001
News	.04	−0.02 to 0.10	1.27	.20
Pornography	.10	0.04-0.16	3.38	.001
Targeted search	.02	−0.04 to 0.08	0.57	.57

## Discussion

### Cross-Sectional Findings

The purpose of this study was to identify Internet-related risk and protective factors for mental health problems and to test if the effects of time spent on the Internet and on various Web-based activities could be accounted for by a number of perceived consequences of those activities. This was investigated by examining the association between adolescents’ general mental health (combined levels of depression, anxiety, and stress or tension) and those Internet-related behaviors, both cross-sectionally and longitudinally over a 4-month period.

The cross-sectional results showed that mental health was predicted by Internet-related behaviors at baseline (15.3% explained variance after adjusting for the number of predictors in the model). Individual effect sizes were rather small (standardized ß=.05-.22). Time spent on the Internet had a larger effect than most individual activities, but consequences of Internet use explained the largest variance in DASS scores (11.1%). Of these, 3 of the 4 negative consequences were the most important predictors (preference for Web-based activities over offline social activities, sleep loss, and withdrawal), whereas the positive consequences were nonsignificant. Internet use that was perceived to increase life meaning or improve school grades or work performance was associated with better mental health, but the effects were smaller than for the negative consequences.

Furthermore, the results showed that time spent on the Internet, social media use, pornography viewing, and school or work activities were only significant predictors when perceived consequences were not accounted for, which suggests that the mental health effects of these activities were explained by the consequences. Web-based gaming, gambling, and targeted searches, on the other hand, were significant predictors of mental health even when controlling for perceived consequences, suggesting that the content of these activities was relatively important in comparison with perceived consequences, with regard to mental health. Together, these results indicate that all Web-based activities measured in this study are predictive of mental health, but only some of them seem to have content-based effects large enough to be detected in a fully adjusted model. The other activities seemed to only affect mental health by means of their perceived consequences, mainly the preference for Web-based interactions, sleep loss, and withdrawal. As these negative consequences are indicative of problematic Internet use [9,14], their relatively strong effect on mental health is expected from a problematic Internet use perspective. It should be noted, however, that perceived consequences may be different from actual consequences.

### Longitudinal Findings

Previous studies have linked sleep loss and withdrawal symptoms to mental health problems and problematic Internet use [9,12,42-45]. The longitudinal analyses in this study similarly suggest that sleep loss and withdrawal (negative mood when content is inaccessible) predict changes in mental health over time (2.1% explained variance), and in fact, these were the only variables to do so in the long term. Longitudinal changes in time spent on the Internet and various activities did not predict change in mental health directly but instead had an indirect effect by predicting changes in sleep loss and withdrawal (3.3% and 6.7% explained variance, respectively). This suggests that time spent on the Internet and content viewed are predictive of mental health mainly because they predict negative perceived consequences, such as sleep loss and withdrawal. This interpretation is in line with the problematic Internet use approach and also supports the differentiation between generalized and specific forms of problematic Internet use (eg, [15-17]), as activities were indeed differently associated with negative consequences. It also suggests that interventions aimed at reducing the negative mental health effects of Internet use could target the negative consequences instead of the Internet use itself. For instance, instead of reducing the time spent on a certain activity, the intervention could focus on making sure that activity does not interfere with sleep. However, with certain types of Internet use, such as gambling, activity-specific interventions may be more effective.

### General Discussion

The results of this study confirm that problematic (or unhealthy) Internet use cannot simply be equated to high-intensity or frequent Internet use. First, although time spent on the Internet was found to be negatively associated with mental health, some activities, such as school work, were positively associated. Second, time spent on the Internet was not an independent risk factor for mental health after accounting for the perceived consequences of Internet use, underlining that Internet use is not intrinsically harmful. Even when it comes to specific activities, for example, gaming, the relationship could be complex. Previous studies have established that gaming has a negative effect on mental health (eg, [12,29]), whereas in this study, the effects were positive. Most studies that have found negative gaming effects have typically only investigated problematic gaming. Thus, it seems possible that gaming has some protective properties when used to a certain extent, but negative consequences might overshadow those properties when used excessively. For instance, in this study, we found that despite its positive mental health effects, gaming significantly predicted sleep loss and withdrawal, which in turn were associated with mental health problems. In line with this, a recent European study on gaming among children aged 6-11 years, found that, once controlled for high usage predictors, gaming was not significantly associated with mental health problems but was instead associated with less peer relationship problems and prosocial deficits [46].

The causal link between general Internet use and mental health also seems complex. Previous authors have acknowledged the possibility that the risk associated with Internet use could reflect an already present disorder, which may have an effect on how the Internet is used [47-49]. Certain cognitive styles that constitute disposition toward using the Internet in certain ways may also influence mental health. For example, Brand et al [50] suggested that problematic Internet use is associated with expectations that the Internet can be used to positively influence mood, which in some cases might be a false assumption on behalf of the user. The disappointing reality of this may in turn worsen preexisting mental health problems. In this study, performing targeted searches (unrelated to school or work) was associated with higher DASS scores and had a larger effect size than any other Web-based activity. A possible explanation for this is that individuals who experience more distress are more prone to use the Internet as a tool for coping with their problems [27]. It could also reflect a general tendency to rely on Web-based sources to solve problems or concerns even when professional help would be more useful. However, because health issues are not the only possible target of Internet searches, future studies will have to explore this hypothesis further.

Furthermore, although Internet-related sleep loss was found to be a longitudinal predictor of mental health, there is an established bidirectional link between sleeping problems and depression [51] as well as mood and affective functioning in general [52]. It therefore seems likely that the relationship between Internet use–related sleep loss and mental health is also reciprocal. Therefore, interventions aimed at reducing problematic Internet use may be more successful if they include simultaneous treatment of comorbid disorders (including depression and sleep disorders). Similarly, a number of previous studies have found problematic gambling to be predictive of generalized problematic Internet use, suggesting that addictive gambling and Internet use have some common etiology [20-23,53]. Our results support this view, as gambling activities were the strongest predictor of perceived withdrawal, suggesting that treatment of problematic Internet use behaviors should also address any gambling problems. However, it is important that future studies examine in more detail which variables act as precursors of harmful Internet use (eg, personality, cognitive, emotional and motivational factors, and existing mental disorders) and which variables act as outcomes and mediators. As certain personality domains might constitute a predisposition toward risk factors such as withdrawal, future studies should investigate the mediating role of such nonpathological variables.

In this study, we found no effect of perceived positive consequences of Internet use on mental health, and it is possible that this is because they are actually rather motives for using the Internet. In other words, participants may have reported consequences they hoped for rather than what actually happened. Sagioglou and Greitemeyer [54] pointed out that self-reported outcomes of different Internet activities may have limited validity, especially when made temporally distant, in which case it may rather reflect what participants see as plausible motivations for their use. More accurate measures may be obtained when participants are asked to rate them immediately after using a Web-based application, which was not possible in this study. Future studies should consider treating positive consequences of Internet use as predictors of using certain Web-based content (in healthy or unhealthy ways) rather than as direct predictors of mental health.

### Limitations

This study is limited by the nature of the measurements used to estimate the participant’s Internet use. One issue of validity concerns the consequences of Internet use, which cannot be assumed to perfectly reflect the real outcomes. In addition to the difficulty of observing the impact of daily activities on one’s own health and behaviors, this measure might also be particularly vulnerable to recall biases and expectancy effects. Hence, this study only intended to measure the perceived consequences. It is also difficult to know whether the perceived consequences are produced by the Internet behaviors or some third factor, such as comorbid disorders. Another limitation of this study is that we did not make in-depth measures of the Web-based content that participants use. Therefore, one should take caution when applying these results to uses of more specific content; for example, different types of games and social networking activities may have different effects on both perceived consequences and mental health. Furthermore, our measurements did not include any problematic Internet use diagnostic tool. It is possible that if we had included more negative consequences of Internet use, or specific problematic Internet use criteria, this would have explained a larger proportion of the effects of the Web-based activities. Finally, there was a notable dropout rate between baseline and follow-up measurements (34%), which reduced the statistical power in the longitudinal analyses compared with the cross-sectional analyses. Also, participation in this study was not completely anonymous, and participants with high suicidal risk were excluded from the data analysis, which could mean that some of the adolescents with the most severe psychopathology were not represented in the analyses.

### Conclusions

Different Web-based activities or content can have specific effects on mental health, even when used in moderate levels and when adjusting for the number of hours spent on the Internet. Web-based activities differ in how consistently, how much, and in what direction they affect mental health. Activities also differ regarding which negative consequences they produce, and those consequences (especially sleep loss and withdrawal) seem to predict mental health outcomes to a greater extent than the activities themselves. Therefore, it seems that time spent on the Internet and Web-based content are predictive of mental health mainly because they predict such negative consequences. These results underscore the importance of differentiating between generalized and specific forms of problematic Internet use. It also confirms that Internet use is not intrinsically harmful, but it depends on the activity that one engages in, and how it affects the individual. Change in mental health over time appears to be best predicted by changes in Internet-related sleep loss and withdrawal, and interventions to reduce harmful Internet use should therefore target such consequences. Positive consequences of Internet use may not predict mental health directly but might predict the propensity to engage in certain Web-based activities excessively or problematically. However, the causality between Internet use and mental health morbidity is complex and likely to be reciprocal, which means interventions or treatments of problematic Internet use might have to be multifaceted to be effective.

## Appendix

Multimedia Appendix 1

## Abbreviations

DASS: Depression Anxiety Stress Scale
DSM: Diagnostic and Statistical Manual of Mental Disorders
IAT: Internet Addiction Test
SUPREME: Suicide prevention through Internet and media based mental health promotion
