# Supplementary material for: Are Mental Health Effects of Internet Use Attributable to the Web-Based Content or Perceived Consequences of Usage? A Longitudinal Study of European Adolescents
Source: JMIR Ment Health. 2016 Jul 13;3(3):e31. doi: 10.2196/mental.5925 (PMC4963606; doi:10.2196/mental.5925)
Supplement: Multimedia Appendix 1 [file mental_v3i3e31_app1.pdf]

## Interventions

Two interventions were used in the SUPREME study (Current Controlled Trials ISRCTN65120704), one full-intervention and one minimal-intervention, both translated and culturally adapted to each participating country. Sixty-nine percent of participants were randomized to the full-intervention group and thirty-one percent to the minimal-intervention group. The full-intervention gave participants access to an intervention website (specifically created for the purpose of the study, which was otherwise closed to the general public) promoting adolescent mental health and suicide prevention by means of improving mental health knowledge and increasing awareness of mental ill-health through informational content. It also offered direct professional support to its users through a chat service. The intervention procedure also consisted of a short presentation (about 10 minutes long) given by the researchers, describing and encouraging use of the website. However, participants' use of the website was completely optional and solely their own initiative. The minimal-intervention comprised of giving each participant a paper leaflet containing contact information for five instances promoting mental health in the participants' country or local area, such as helplines, youth centers and websites. Services included on the leaflet were determined by the individual centers.

While all participants in both conditions received the minimal-intervention leaflet after baseline data collection, only participants in the full-intervention group received the additional presentation about the website. Participants were blind to which condition their school had been assigned and were not informed about the existence of the other experimental condition. The website presentation and the minimal-intervention were administered again at two months, following the second wave of data collection. The study employed a waiting-list design in which participants in the minimal-intervention condition were granted access to the intervention website after the third wave of data collection. In the present study, the differences between the two intervention groups were controlled for in the statistical analyses.

## Secondary statistical analyses

The cross-sectional regression analysis in this article was calculated to predict raw DASS-42 scores (i.e. unstandardized, non-transformed scores). Because most subjects were relatively healthy, the distribution of scores were positively skewed (*skewness* = 1.582; *kurtosis* = 2.855; Kolmogorov-Smirnova test of normality: $P < .001$), and thus violated the statistical assumption that the dependent variable is normally distributed. To investigate the impact of this violation on the reliability of the results, the scores were transformed to approach normality, after which the analysis was re-run. Various transformations were tested as suggested by Tabachnick and Fidell [1]. Transformation by taking the square root of the raw scores proved to be the best solution, and although the Kolmogorov-Smirnova test of normality was still significant ($P < .001$), the distribution approximated normality (*skewness* = 0.362; *kurtosis* = 0.002) and was considered an improvement (this consideration was validated by visual inspection of P-P plots, Q-Q plots, and scatterplots of regression standardized residuals). The cross-sectional regression analysis was therefore re-run using this transformed variable. The results obtained through this analysis resembled the original results to a high degree, with no major differences in effect sizes or p-values that would substantially invalidate the previous analysis. Therefore, we concluded that the primary regression analysis was robust enough to be reported in its original form. Regarding the longitudinal regression analysis, change in DASS scores between T1 and T3 was not substantially skewed (*Skewness* = -0.421), thus transformation of this variable was considered unnecessary.

## References


1. Tabachnick BG, Fidell LS. 2007. *Using Multivariate Statistics*. 5th edition: pp. 89. Boston, MA: Pearson Education/Allyn & Bacon. ISBN:0-205-45938-2
